# Bioluminescence as a functional tool for visualizing and controlling neuronal activity *in vivo*

**DOI:** 10.1117/1.NPh.11.2.024203

**Published:** 2024-02-12

**Authors:** Montserrat Porta-de-la-Riva, Luis-Felipe Morales-Curiel, Adriana Carolina Gonzalez, Michael Krieg

**Affiliations:** ICFO—Institut de Ciències Fotòniques, The Barcelona Institute of Science and Technology, Castelldefels, Barcelona, Spain

**Keywords:** optics, photonics, light, optogenetics, bioluminescence, neuroscience, *Caenorhabditis elegans*, luciferases

## Abstract

The use of bioluminescence as a reporter for physiology in neuroscience is as old as the discovery of the calcium-dependent photon emission of aequorin. Over the years, luciferases have been largely replaced by fluorescent reporters, but recently, the field has seen a renaissance of bioluminescent probes, catalyzed by unique developments in imaging technology, bioengineering, and biochemistry to produce luciferases with previously unseen colors and intensity. This is not surprising as the advantages of bioluminescence make luciferases very attractive for noninvasive, longitudinal *in vivo* observations without the need of an excitation light source. Here, we review how the development of dedicated and specific sensor-luciferases afforded, among others, transcranial imaging of calcium and neurotransmitters, or cellular metabolites and physical quantities such as forces and membrane voltage. Further, the increased versatility and light output of luciferases have paved the way for a new field of functional bioluminescence optogenetics, in which the photon emission of the luciferase is coupled to the gating of a photosensor, e.g., a channelrhodopsin and we review how they have been successfully used to engineer synthetic neuronal connections. Finally, we provide a primer to consider important factors in setting up functional bioluminescence experiments, with a particular focus on the genetic model *Caenorhabditis elegans*, and discuss the leading challenges that the field needs to overcome to regain a competitive advantage over fluorescence modalities. Together, our paper caters to experienced users of bioluminescence as well as novices who would like to experience the advantages of luciferases in their own hand.

## 1 Introduction

**Fig. 1:**
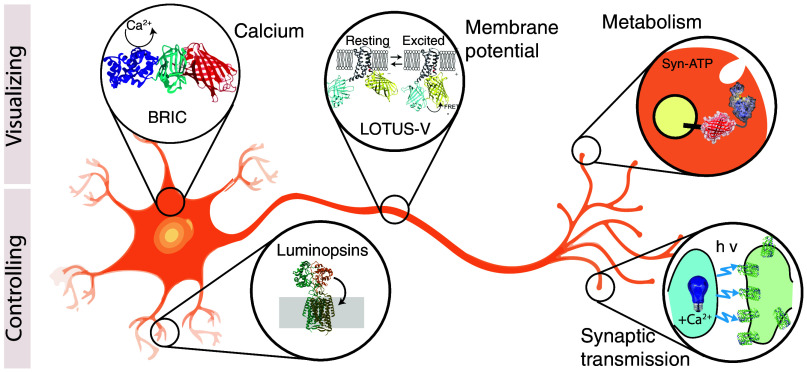
Different luciferases and their application in neurons as sensors and controllers of neuronal activity. Graphical representation of how bioluminescence can provide insight into biosensing and controlling neuronal activity. Upper panel: different constructs have been engineered that are sensitive to calcium, membrane potential, or synaptic ATP consumption. Lower panel: the functional coupling of light emission and light-sensitive proteins has been employed to control membrane voltage and synaptic transmission between two neurons in a living animal (see text for references).

Bioluminescence is the natural ability of certain organisms, such as fireflies, and some marine organisms, to produce light through the enzymatic oxidation of a metabolic cofactor by a luciferase or photoprotein. Since their first discovery in the genus *Aequoria*, luciferases have become a popular tool in biotechnology and bioengineering because they can be genetically encoded in any organism and for their unique property to emit light in response to external inputs—in nearly complete absence of any background signal. This afforded the development of ultrasensitive bioassays with luciferases as sensors of metabolic activity reaching attomolar sensitivity^1^ but also to drive cellular processes in conjunction with light-sensitive proteins.^2^ To date, various bioluminescent sensors and actuators have been targeted to neurons, not only with the aim of monitoring changes in cellular physiology, such as calcium concentrations, ATP consumption, and membrane voltage, but also to activate neurons and re-engineer neuronal connections (Fig. 1).^3^ The concomitant increases in light emission upon solute or metabolite binding can be conveniently recorded on a camera or a photodiode. This noninvasive and highly sensitive imaging method allows scientists to record various biological phenomena in cellular and molecular neuroscience,^4^^,^^5^ providing valuable insights into complex biological processes while minimizing harm to the subjects under study. Bioluminescence imaging has become a powerful technique that is extensively used in neuroscience to visualize and study both physiological and pathological processes within living organisms. The applications in the field have expanded in the recent years, with more powerful luciferases/cofactor systems emerging that afford biosensing deep inside the tissue and even deploy control on the activity of neuronal circuits (Fig. 1).

### 1.1 Luciferases as Background-Free Cellular Labels for Deep Tissue Imaging

**Fig. 2:**
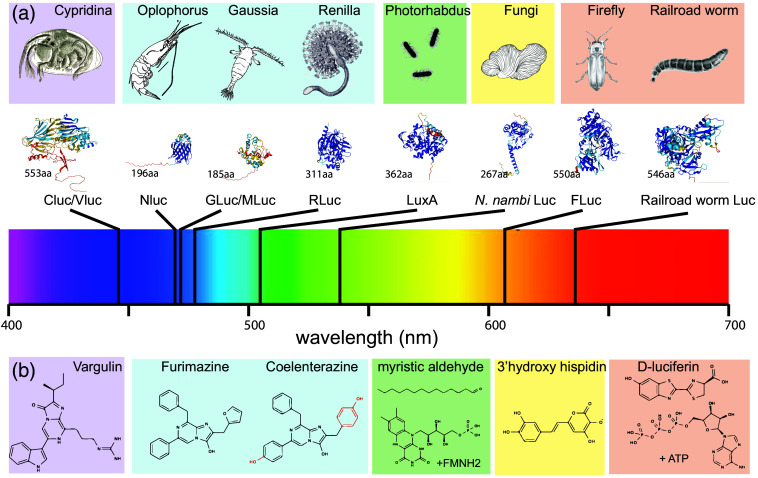
Overview of most common luciferases and their cofactors used in neuroscience applications. (a) Diversity of bioluminescent organisms and their luciferases. The shading indicates the class of cofactors and their derivatives employed: purple = vargulin, blue = coelenterazine, green = FMNH2/aldehyde, yellow = hispidine, and orange = d-luciferin. The catalytic subunits of the luciferase have been modeled in AlphaFold2 and are scaled approximately to their relative size. The number of aminoacids is indicated in each structure, and their peak emission wavelength is visualized below as a vertical black line within the visible spectrum. (b) Chemical structures of some representative luciferin substrates used by the luciferases mentioned in panel (a). The display is not exhaustive and various chemical modifications exist for CTZs, d-luciferins, and others. Some luciferins require secondary substrates for their activity (FMNH2, reduced Flavin-mononucleotide; ATP, adenosine triphosphate), others require calcium as cofactor (e.g., aequorin, not shown), and most if not all require oxygen for their reaction.

More than 30 different luciferase/luciferin pairs are known to date.^1^^,^^6^^–^^8^ Many of these enzymes and their cofactors have been characterized,^1^ and found to differ in emission wavelength, protein size or type, and chemistry of the cofactor (Fig. 2). The most commonly applied luciferases have been derived from firefly (Fluc), bacteria (luxABCDE), and several marine organisms such as *Cypridina noctiluca* (Cluc), *Renilla reniformis* (Rluc), *Gaussia princeps* (Gluc), and *Oplophorus gracilirostris* (Oluc/Nluc).^6^ Biotechnological applications are dominated by the firefly luciferase from *Photinus pyralis*, albeit it requires ATP as an additional cofactor to the oxidation of a substrate. The yellow emission spectrum ($\lambda_{\mathrm{Em}}\approx 560\;\mathrm{nm}$) of firefly luciferase and its variations make them highly advantageous for applications within living organisms compared with blue emitting luciferases from marine organisms. Because biological tissues demonstrate minimal absorption and scattering at longer wavelengths, emitted photons can penetrate deep into tissue.^9^^,^^10^ This effectively affords the visualization of neurons inside deeper layers of the brain, which are inaccessible with common fluorescence microscopy because of the high light-intensity required for excitation and its adverse effects. Thus bioluminescence microscopy is a promising tool for transcranial neuronal activity imaging.^6^ Further, the number of emitted photons can be quantified, allowing a comparison between conditions and longitudinal studies aimed at understanding cellular physiology.^11^ An additional and important advantage of bioluminescence is the lack of artifacts that are common in fluorescence imaging such as phototoxicity, background autofluorescence, and fluorophore bleaching.^12^^,^^13^ Still, photons can be absorbed or scattered, which limits the tissue penetration of the emitted light and thus the depths and resolution at which bioluminescence can be recorded.

In an effort to increase tissue penetration, protein and chemical engineers generated luciferases with a farther red-shifted emission spectrum. To access the optical window in biology (a region in the electromagnetic spectrum between 650 and 1350 nm, where light has its maximum depth of penetration in tissue), researchers created a synthetic luciferase/luciferin combination called AkaBLI.^14^ This novel system, built upon Fluc, boasts a peak emission at 650 nm. To showcase the superior performance of AkaBLI in animals, the new combination was used to image cFOS activity in the CA1 hippocampal region in freely behaving mice challenged with a new environment.^14^ This improved system provided up to 1000-fold enhancement over conventional firefly luciferases that can be used to directly monitor much fewer ensembles of neurons than many other noninvasive optical methods. Strikingly, the number of photons emitted per time from AkaBLI in isolated cells is only slightly higher than that from the conventional firefly/D-luciferin system,^15^ suggesting that tissue penetration of the red-shifted AkaBLI, rather than intensity is what indeed makes the synthetic system more powerful for *in vivo* imaging.^10^

However, firefly luciferases and their derivatives suffer from a low catalytic activity, that, despite their high quantum yield (the probability at which oxidation of the substrate leads to photon emission), leads to a low photon output. Consequently, imaging live cells and animals^4^ required long exposure times in the tens of second or even minute scale. This limitation restricted the use of firefly luciferases primarily to questions that did not go beyond long-term reporter assays.^5^^,^^16^^–^^19^ Thus, the ideal luciferase for *in vivo* applications would combine a red-shifted emission spectrum with a high photon emission intensity.

### 1.2 Toward Brighter Luciferases for Applications in Neuroscience

**Fig. 3:**
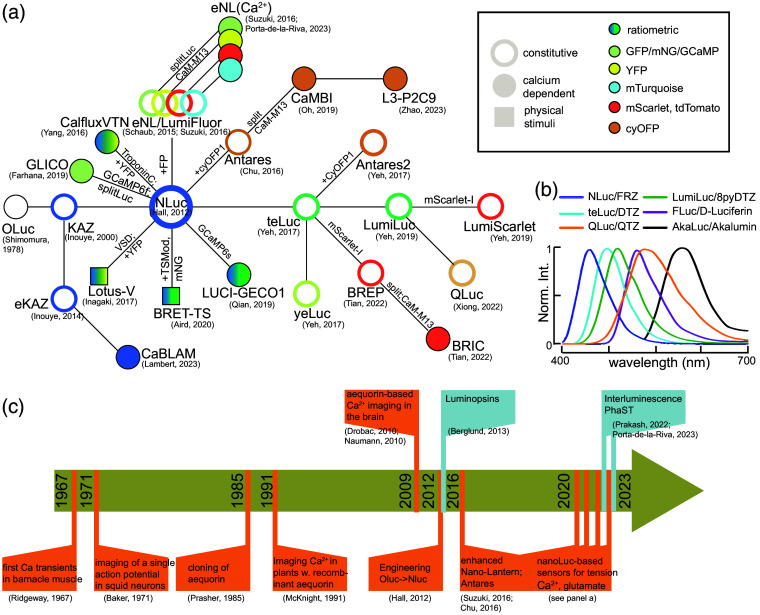
Bioluminescent sensors of calcium and physical parameters as promising alternatives to fluorescent sensors in neuroscience. (a) Lineage of the Oluc/Nluc-based sensors of calcium, membrane potential, and mechanical tension. Kaz is the 19 kDa catalytic subunit of the Oluc luciferase.^23^ Nluc is also referred to as nanoKaz in its codon-optimized form.^24^ LumiFluor is a c-terminal fusion of Nluc and an FP (GpNLuc for eGFP^25^). Empty circles indicate constitutive luciferases with colors indicating wavelength of the emitted photons, filled circles indicate ${\mathrm{Ca}}^{2+}$ sensors, and squares denote sensors of physical parameters. (b) Normalized emission spectra for different engineered luciferase/luciferin systems. Note, different systems have different photon output, with the teLuc/DTZ system ≈120× more than Fluc/d-luciferin, 2× more than NLuc/Furimazine and QLuc/4-quinolinylterazine (QTZ) more than 11× more photons than AkaLuc/Akalum between 600 and 700 nm. Graph adapted from Ref. 26. (c) Milestones in the development of bioluminescent proteins as biosensors (orange flags) and actuators (blue flags).

Fortunately, the engineering of small, powerful luciferases from marine organisms, especially from *O. gracilirostris* (Oluc) has sparked new optimism in expanding the applications of bioluminescence^20^^,^^21^ that require subcellular resolution or subsecond cellular dynamics^22^ inside animals [Fig. 3(a)]. Oluc is a secreted protein complex of 110 to 130 kDa^27^ that emits a bright intense 460 nm flash of light with broad substrate specificity but without the requirement for any other cellular cofactors such as ATP. Follow-up investigations determined that the bioluminescent function within the 110 kDa enzymatic complex is specifically confined to a 19 kDa subunit, known as Kaz. This discovery marked Kaz as one of the most compact catalytically active luciferases.^23^ Several rounds of rational and random mutagenesis on the wildtype form of this 19 kDa protein stabilized its structure and activity [Fig. 3(a)], obtaining one of the brightest genetically encoded light generators to date.^21^^,^^28^ The optimization went beyond protein engineering, involving also the discovery and synthesis of a novel substrate, furimazine, with the aim to replace the native substrate, coelenterazine (CTZ), which is unstable and prone to spontaneous decomposition *in vivo*, in addition to bearing residual autoluminescence.^28^ A total of 16 mutations on Kaz lead to the well known NanoLuc (Nluc; the codon-optimized form is called nanoKaz^24^), which proved more than 2.5 million-fold brighter compared with the parent wildtype protein and 150-fold than firefly luciferase in conjunction with the optimized furimazine cofactor.^28^ Additionally, and very important for many applications in live cells, the Nluc is ATP-independent and thus does not pose additional metabolic burden on the host cells. The optimization of enzyme and cofactor chemistry resulted in a higher photon emission with increased quantum yield. For example, the quantum yield of the Nluc reached 28% (compared with 40% for Fluc) and is thus several fold higher than other marine luciferases such as Rluc, which suffers from a low quantum yield of only 2% to 5%.^28^^–^^30^ Interestingly though, in nature, Rluc associates with a green fluorescent protein (GFP) in a highly specific manner^31^ that accepts the excited state energy through nearly perfect bioluminescence resonance energy transfer (BRET)^32^ from the oxyluciferin and emits a photon through fluorescence, resulting in an ≈3× higher quantum yield of 13%.^33^ This mechanism is not uncommon, as many marine organisms take advantage of energy transfer from the luciferase moiety to fluorescent proteins (FPs) to considerably increase the bioluminescent quantum yield. This works as long as the quantum yield of the FP is higher than that of the oxidized substrate. Having this recognized, this concept was applied to synthesize artificial luciferases through fusion proteins between the luciferase moiety and an acceptor fluorophore to increase the total quantum yield and thus their brightness. This approach was first applied to generate Rluc8-Venus with fivefold greater light output than Rluc8 (coined Nano-Lantern^34^^,^^35^), which afforded subcellular imaging of organelle dynamics. Still, Rluc has a very low quantum yield and produces less than 10 photons/s/molecule. Several groups hence applied this approach to the powerful Nluc, and increased the brightness through improved quantum yield of the resulting luminescent proteins, by attaching the luc moiety to FPs, a class of probes that were confusingly called “enhanced Nano-Lantern (eNL),”^22^ “LumiFluors,”^25^ or “Antares”^36^ [Fig. 3(a)]. With these improvements of luciferases and their cofactors (Box 1), bioluminescence imaging entered the microscopy realm, offering new possibilities to the investigation of subcellular structures with high spatial resolution.

**Box 1:** Luciferases and their properties

Luciferases catalyze the oxidation of a substrate (generally termed luciferin) and the reaction may emit light when the electronically excited oxoluciferin relaxes to its ground state, with a probability described as the quantum yield. Many different variants exist and their capability to produce light evolved independently multiple times in bacteria, dinoflagellates, arthropods, mollusks, annelids, echinoderms, urochordates, and even vertebrates.^9^
*Cofactors*. Even though 11 luciferase/luciferin pairs have been characterized (out of more than 30 discovered so far), most applications take advantage of luciferases oxidizing d-luciferin or CTZ and their derivatives.^1^ The chemical properties of the substrate not only determine the affinity, quantum yield, and wavelength of the emitted light, but also the effective availability in biological cells.^37^ d-luciferin and CTZ differ in their water solubility, with CTZ and its derivatives furimazine and hikarazine being generally more hydrophobic. Several CTZ analogs have been engineered to increase their bioavailability,^1^^,^^7^ specifically to overcome delivery challenges across the blood–brain barrier.^38^^,^^39^
*Wavelength selectivity.* The emission wavelength of the different luciferase/luciferin systems ranges from 450 to 700 nm [Fig. 2(a)]. In addition to the specific cofactor, intermolecular interactions and solvent polarity also determine the emission.^37^ The choice of the right wavelength is important for the application. For combining multiple luciferases, the emission spectra should be as narrow as possible (e.g., Nluc, which has a 20 nm narrower spectrum than Rluc^28^), red-shifted is convenient for deep tissue imaging,^7^ and in combination with photosensitive proteins, emission needs to have good overlap with their action spectrum, e.g., of channelrhodopsin. The wavelength of the emitted light largely depends on the type of luciferin, and different luciferases have been engineered to accept cofactors that produce large red shifts (e.g., Qluc + QTZ^26^). Red-shifting can also be achieved by fusing the luciferase moiety to various FPs.^22^^,^^36^
*Kinetics.* Together with their luciferin cofactor, different luciferase complexes show different kinetics, which can be generally classified as flash or glow type luciferase, depending on whether they emit light rapidly or steadily. The kinetics of constitutive luciferases is limited by substrate turnover, availability in the tissue but also through covalent inhibition by a derivative of the CTZ cofactor during the reaction with *Gaussia* but not with Nluc.^40^ Often, a choice has to be made and it is more convenient to trigger as much photons as possible per unit time, or to produce a steady emission over periods of many hours. This can either be achieved by spatiotemporally controlled perfusion of the luciferin or split luciferases that reconstitute activity upon binding to secondary metabolites, such as calcium ions (${\mathrm{Ca}}^{2+}$) or cyclic adenosine monophosphate. Alternatively, flash or glow type luciferases differ in their photon production rates. Despite many years of research, their specific reaction mechanism leading to various color production described from the same substrates is still being worked out.^41^^–^^43^

### 1.3 Optimizing Emission Wavelength for *in Vivo* Neuroscience and Brain Imaging

In the quest to generate powerful luciferases for *in vivo* imaging, the Nluc required emission wavelengths that are further red-shifted, to avoid scattering and photon loss due to absorption.^10^ The fusion of the luc to the FP not only afforded an increase in quantum yield, but also a possible shift in emission wavelength [Fig. 3(a)]. Thus, a fusion of the Nluc moiety with tdTomato improved emission in the 600 nm range but also retained a considerable emission peak at 450 nm, probably due to a poor overlap between the emission spectrum of the luc moiety and the excitation spectrum of the FP.^22^ With the aim to increase the overlap and thus BRET efficiency, Nluc was combined with cyan-excited orange FPs, LSSmOrange (e.g., OgNLuc)^25^ or CyOFP1 (e.g., Antares),^36^ two long Stokes shift FPs with broad excitation between 450 and 500 nm and red-shifted emission. Even though the OgNLuc resulted in a better match between the emission of the luc and absorption of the FP, the CyOFP1 in Antares has a very high quantum yield of 0.78. However, to compensate for the suboptimal match between Nluc emission and CyOFP excitation, BRET efficiency in Antares was increased with two copies of CyOFP1, fused to both the N- and C-termini of the Nluc.^36^

Enzymes exhibiting improved red-shifted emission were also generated by subjecting the Nluc luciferase moiety to random mutagenesis. This resulted in the development of yeLuc and teLuc [Fig. 2(b)], showcasing peak emissions at 527 and 502 nm, respectively, along with a twofold increase in quantum yield. This red shift, however, required novel substrates: selenoterazine (STZ) and diphenylterazine (DTZ), respectively.^44^ Similar to Antares, the fusion of teLuc with CyOFP1 enabled a luciferase with higher quantum yield (termed Antares2) and generated up to 60 times more photons than firefly luciferase at 600 nm.^44^ However, it was subsequently shown *in vivo* that the full potential of Antares2 is not reached due the limited bioavailability and stability of the substrate.^14^ To overcome the challenging cofactor administration *in vivo*, a fluorinated furimazine (FFz) was synthesized and found to achieve better photon counts, due to better stability in aqueous solution and bioavailability.^45^ Further rounds of mutagenesis of the luc moiety led to yellow (LumiLuc^46^) and amber emitting variants (QLuc^26^) [Fig. 3(a)], pushing the luciferase activity wide into the red spectrum, even in absence of a fluorescent protein fusion [Fig. 2(b)]. These variants were shown to partially overcome the limitation of Nluc compared with Fluc for deep tissue imaging and provided a brighter signal when targeted to the liver in compared to conventional Gluc-d-luciferin imaging conditions transgenic animals.^46^ Notably, as the photons are produced from the excited state during the oxidation of the substrate, these engineered luciferases require their own chemically engineered cofactor (e.g., teLuc + DTZ, yeLuc + STZ, LumiLuc + 8pyDTZ, and QLuc + QTZ^26^^,^^38^^,^^44^) for the wavelength shift, but might cross react with furimazine to produce blue photons.^26^ Taken together, many different luciferase/luciferin systems^1^ have been engineered and their advantages need to be assessed prior to each experiment taking into account differences in power, wavelength, substrate solubility, and bioavailability.

## 2 Functional Imaging of Physical and Physiological Properties with Bioluminescence Microscopy

Owing to the nearly absent background luminescence, luciferases afforded some of the most sensitive biosensors for *in vitro* and *in vivo* studies.^8^ Even though the quest to produce the best and photon-richest enzymes with new spectral properties is ongoing, there is a large diversity of different bioluminescent indicators and sensors for various purposes, ranging from intracellular sensing of ATP to neurotransmitter secretion and ion indicators (Fig. 1). Given Nluc’s demonstrated advantages in size, stability, and photon output among other known luciferases, this section will primarily center on sensors that were derived from it [Fig. 3(a)].

### 2.1 Imaging Calcium Activity with Luciferases

Calcium is of critical importance to neurons as it participates in the transmission of the depolarizing signal and contributes to synaptic activity.^47^ Consequently, significant efforts have been dedicated to measuring calcium ion concentrations within cells, particularly in neurons. Before the invention of fluorescent chemical calcium indicators, the endogenous calcium-dependent activity of aequorin was exploited to image fluctuations in calcium in live cells in general and neurons in particular.^47^ First, aequorin purified from jellyfish was microinjected into cells that were large and robust enough to survive such treatments, such as the barnacle muscle^48^^,^^49^ or giant squid axon.^50^ These experiments using bioluminescent probes constituted the first reliable measurements of intracellular calcium [Fig. 3(c)]. Remarkably, it was possible to obtain a luminescent response of aequorin to a single action potential^51^—by averaging 1500 recordings. After molecular cloning of aequorin,^52^ the cDNA could be introduced into various heterologous cell types and expressed in subcellular compartments to visualize calcium entry in plants upon mechanical touch,^53^ neuronal nuclei or mitochondria,^54^ and single neurons in neocortical brain slices.^55^ It also afforded the quantification of calcium dynamics in freely behaving zebrafish larvae^11^ using single photon counting over the course of 1 h. However, due to the fast pace in laser technology and molecular engineering, the advantages of chemical, fluorescent indicators of calcium ions outweighed the few advantages afforded by the poor photon emission and ion selectivity of aequorin.^56^

The discovery of the Nluc not only led to the smallest and brightest genetically encoded light generator to date but also brought the possibility to engineer powerful luminescent calcium sensors [Fig. 3(a)]. In one of the first iterations, a calcium-sensitive eNL was constructed by inserting a well-defined calcium-binding motif composed of calmodulin (CaM) and the M13 peptide into the split Nluc moiety. Importantly, in presence of high calcium concentrations, the split luciferase reconstituted catalytic activity and photon output, making these eNLs a versatile intensiometric calcium sensor. The eNL with mTurquoise and mNeongreen as acceptors worked particularly well and showed video-rate calcium dynamics in cultured cells and transgenic *Caenorhabditis elegans* animals.^12^^,^^22^ Several variants with different calcium affinity were optimized to visualize calcium in subcellular organelles such as the endoplasmatic reticulum and mitochondria.^57^ A ratiometric BRET sensor (termed CalfluxVTN) was developed in which the Nluc moiety and a Venus fluorophore were connected by a troponin-C calcium sensor with optimized linker residues to maximize dynamic range.^58^ Calcium binding to troponin-C induces a conformation change that bring the luc and FP into BRET distance, thus changing the emission of the FP. This sensor was successfully applied to cultured hippocampal neurons and organotypic slices to visualize optogenetically and chemically induced calcium transients.^58^ Leveraging the superior performance of recent fluorescent calcium indicators, several labs engineered a fusion of either constitutive^59^ or split Nluc moiety with GCaMP6 (GLICO^60^). While the constitutively active Nluc enables ratiometric recording of the luc-emitted and the GCaMP6s emitted photons, the calcium-independent activity of Nluc generates large background of out-of-focus light, which degenerates the signal in 3D environments such as the brain.^59^ Conversely, the combination of a split Nluc with GCaMP6f provided a dynamic range of 2200% and is thus a promising candidate for visualizing and quantifying calcium dynamics *in vivo*.^60^

In the pursuit of enhancing bioluminescent calcium recording within deep tissues, researchers engineered calcium sensitivity into Antares. They achieved this by integrating the CaM-M13 peptide into the Nluc component, resulting in the development of a novel sensor called orange calcium-modulated bioluminescent indicator (Orange CaMBI).^61^ The improved red shift facilitated imaging of calcium dynamics in the liver and, together with the novel substrate cephalofurimazine (CFz), allowed delivery across the blood/brain barrier, affording transcranial imaging of neuronal activity in awake mice.^39^ As Nluc requires oxygen for light generation, a potential complication of using these enzymes and their derivatives in the brain comes from the hemodynamic response secondary to neuronal activity. The resupply of oxygen might cause a variation in luminescence levels, which could be misinterpreted as variations in neuronal activity. Interestingly, the authors could successfully decouple the immediate early increase of luminescence due to calcium and the lagging increase due to hemodynamics. Further, the luminescence imaging in awake mice showed a bilateral asymmetric response, due to the primary sensory processing and pan-cortical arousal response.^39^

Another red-shifted calcium indicator was made from a split teLuc fused to the red FP mScarlet-I (BRIC, bioluminescent red indicator for Ca^2+^).^38^ Strikingly, BRIC oxidation of its synthetic substrate ETZ was able to report fear-induced activity in the central amygdala and hippocampal activity in response to kainate acid in awake mice, where the combination of Orange CaMBI and FFz did not show detectable luminescence.^38^^,^^61^

### 2.2 Physical Stimuli

#### 2.2.1 Membrane potential in neurons and mitochondria

Contrary to the abundance of bioluminescent calcium indicators, membrane potential indicators based on bioluminescence are just emerging. One of the difficulties in designing and deploying voltage sensors to neurons in living animals is the requirement for fast response time and increased recording speed to resolve individual action potentials. The Nagai lab developed a luminescent optical tool for universal sensing of voltage consisting of the voltage sensing domain (VSD) of the *Ciona intestinalis* ascidian and the Nluc-Venus BRET pair which was used to record transient membrane depolarizations due to KCl in PC12 cells with 30 Hz frame rate.^62^ Even though this speed is too slow for counting action potentials, it is impressively fast and could facilitate recordings of receptor potentials or graded membrane depolarization in animals lacking action potentials, such as *C. elegans*. As the authors pointed out, recording voltage with bioluminescent dyes not only facilitates background free imaging and simultaneous voltage recordings during optogenetic stimulation, but also enables long term imaging by minimizing phototoxicity related to the high excitation intensities that are commonly required with some genetically encoded fluorescent indicators of membrane voltage.^63^ Recently, a voltage indicator was produced from the bacterial Lux system that does not require the addition of exogenous luciferin, due to coexpression of the enhanced luxAB luciferase (eluxAB) with the luciferin-producing genes (luxCDE). In the resulting AMBER, the luciferase eluxAB was fused to the Ciona VSD and the YPet FP, reversibly activating enzymatic activity as a function of membrane voltage.^64^ The voltage domain was also fused to the cytoplasmic flavin reductase, which produces FMNH2 as necessary cosubstrate. Even though the eluxAB luciferase was codon optimized for better expression, the photon output from bacterial luciferases is generally weak and, even when performing better than the non-enhanced version, AMBER is not an exception. In addition, it requires FMNH2 as well as the bacterial fatty acid reductase complex (luxCDE), consuming an important cellular metabolite and producing a potentially toxic fatty aldehyde as an intermediate product.^65^ Nevertheless, because AMBER sustains luminescence without exogenous delivery of a cofactor, it holds promise to overcome the challenges in cofactor delivery in animals with an exoskeleton. Along these lines, AMBER was successfully expressed in the *C. elegans* pharynx and touch receptor neurons, where it showed bursts of activity while making spatially restricted movements and frequent reversals.^64^

A different approach has been used to image membrane potential in mitochondria. In this case, a caged luciferin cofactor is taken up into the mitochondrial matrix, where it reacts with a second uncaging reagent through click chemistry.^66^ Both chemicals are efficiently imported into healthy mitochondria via a modification with a positively charged triphenylphosphonium group, showing approximately 100 and 1000 times greater accumulation in the mitochondria compared to the cytosol or extracellular matrix.^67^ Because the luciferin becomes only accessible after it passed through the mitochondria, this approach was termed MAL for mitochondrial-activated luciferin. Once inside, the protected luciferin is uncaged and diffuses out of the mitochondria where it gets oxidized by cytosolic luciferases. As the import of positively charged chemicals into mitochondria depends on an intact voltage gradient across the inner-mitochondrial membrane, the luminescent levels are hence an indirect readout of the membrane potential.^66^

#### 2.2.2 Membrane tension and mechanical stress

The quantification of mechanical tension in intact organisms is a formidable challenge and requires sophisticated technology^68^^,^^69^ or genetically encoded sensors.^70^ A dedicated BRET tension sensor (TS)^71^ adds to the toolbox and potentially alleviates some of the limiting challenges in existing fluorescence resonance energy transfer (FRET) TS.^70^^,^^72^ In the absence of tension, the unloaded BRET sensor boasts a robust ≈60% apparent BRET efficiency upon addition of Nluc’s chemiluminescent substrate, furimazine, whereas the unloaded TSMod under the same conditions has only 25% FRET efficiency. These differences may relate to the larger size of the donor fluorophore, which limits the equilibrium FRET distance, but also to the larger spectral separation between the Nluc and the acceptor in the BRET-TS construct. Thus, together with the usual benefits of bioluminescence imaging, BRET-TSs have the additional advantage of a higher dynamic range and force resolution. As no excitation laser is required, no acceptor cross-excitation by the donor laser, a common problem in sensitized emission FRET,^70^ is expected. Importantly, the BRET-TS was able to report tension gradients in peripheral focal adhesions when embedded into vinculin, with an average of 31% variation in BRET levels across peripheral focal adhesions, where previous studies reported FRET gradients of 5%.^73^ This, however, required exposure times ranging from 15 s to 2 min.^71^

### 2.3 Luciferases as Sensors for Synaptic Function

The notion that firefly luciferase depends on ATP for photon production can be harnessed as a background-free sensor to measure ATP levels inside cells and the extracellular matrix.^74^^,^^75^ Intriguingly, the number of photons emitted is directly proportional to the number of ATP molecules consumed, providing a quantitative readout for metabolic activity. This property of Fluc as an ATP sensor was harnessed to quantify the metabolic burden of synaptic activity^4^ and the resting presynaptic vesicle pool in nerve terminals^76^ and provided exciting insight into the physiology of synapses. These quantitative measurements of ATP consumption at the synapses were made with *Syn-ATP*, a thermo-stable firefly luciferase used in mammalian cells, fused to mCherry as a ratiometric indicator and synaptophysin as a synaptic targeting signal. To provide a quantitative readout, Syn-ATP was calibrated *in situ*, on permeabilized cells with known concentrations of ATP. These careful calibrations showed that each synaptic bouton contains $10^{6}$ molecules or 1.4 mM of ATP, which is consumed by action potential driven synaptic activity.^4^ With further measurements in metabolically active and inactive neurons, the authors could show that electrical activity induces ATP synthesis, which is required for energy-homeostasis in active neurons.^4^

The same sensor revealed insights into the metabolic expenditure of neurons at rest. The resting pool of synaptic vesicles at nerve terminals displays a large proton gradient used to import neurotransmitters into the vesicle lumen. This proton gradient is established and maintained by vesicular ATPases (v-ATPases) that transport 3 protons for each hydrolyzed ATP. Pulido and Ryan^76^ recently found that the proton gradient slowly dissipates, which requires a constant ATP consumption through v-ATPases activity to restore the gradient. Surprisingly, more that 44% is spent by the v-ATPases that generate a proton gradient across the vesicular membrane, and only little ATP is spent by the axonal ${\mathrm{Na}}^{+}/K^{+}$ pump to maintain the resting membrane potential. Leveraging the quantitative aspect of bioluminescence measurement, it was demonstrated that 3100 ATP molecules are expended per second at each nerve terminal. These experiment revealed the surprising fact that even synaptic inactivity requires a constant energy input.^76^

In addition to sensors for calcium ions and ATP, bioluminescent sensors that detect secreted neurotransmitters provide a spatial map of neuronal signaling. Unlike sensors for calcium and membrane potential, extracellular sensors for synaptic transmitters directly reveal successful neuronal signaling. One of the first bioluminescent indicators for glutamate, bioluminescent indicator of the neurotransmitter glutamate (BLING), consists of the Nluc moiety fused to the glutamate binding protein Glt1, that is anchored to the cell surface through a PDGF-receptor transmembrane domain.^77^ BLING reports a 10% luminescent change in concentrations of 10  μM glutamate. It was successfully expressed in the mouse sensory cortex, where it reported higher levels of glutamate release upon chemically induced seizures.^77^ Like other bioluminescent sensors, their background and signal-to-noise ratio (SNR) is superior to fluorescence measurement and could potentially resolve synaptic transmission events inside deep brain tissues. However, the poor photon output is incompatible with high spatial resolution and fast circuit dynamics. Alternative bioluminescent sensors exist for different neurotransmitters. For instance, in the case of norepinephrine, these sensors are structured using a chemically shielded form of luciferin. Upon encountering norepinephrine, a specific chemical reaction occurs, removing this shield from the luciferin. Subsequently, the luciferase enzyme can then oxidize the exposed luciferin, generating light as a measurable response.^78^

Taken together, bioluminescent strategies for the detection of metabolites and signaling molecules are becoming increasingly popular. This is due to the ever-expanding palette of enzymes, colors, highly efficient microscopy techniques and extensive genetic methods. For a comprehensive list of other bioluminescent indicators, the reader should refer to Ref. 79.

## 3 Functional Control over Neuronal Activity with Bioluminescence

Due to the complexity of the brain with the wiring of excitatory and inhibitory neurons within the same or adjacent circuits, evidence is mounting that broad, non-specific stimulation of certain brain region does not have the optimal outcome.^80^^,^^81^ Therefore, emerging technologies are currently being developed to facilitate neuromodulation that is specific to cell types and neural circuits. Genetically sensitizing the target cells to optical,^82^ magnetic,^83^ or ultrasound^84^ stimulation is gaining popularity due to emerging solutions in gene therapy and viral delivery.^85^ By harnessing the power of light-sensitive proteins called opsins (see Box 2), optogenetics enables precise control of cell activity with millisecond precision, allowing researchers to manipulate and monitor biological functions in a highly targeted manner.^86^ This innovative technique has opened up new avenues for understanding the complexities of the nervous system, and offers potential therapeutic targets for treating neurological and psychiatric disorders. Still, one of the difficulties, and probably the limiting factors for translating this technology from the bench to the bedside, is the challenge associated with bringing the stimulating light in vicinity of the opsin.^87^ In this context, bioluminescence has a great potential for cell or even synapse specific modulation^3^ and could possibly overcome the light delivery challenge to deep regions in the brain.^2^

**Box 2:** Photosensitizers for functional bioluminescence optogenetics

*Rhodopsins* are a diverse group of light sensitive transmembrane proteins that obtain their intrinsinsic photosensitivity from a covalent, prosthetic retinal group. While animal rhodopsins (type 2) are G-coupled receptors that trigger visual phototransduction, type 1 rhodopsins have a wide variety of functions and have been described in organisms as diverse as an archaea, bacteria, and algae. The most common rhodopsins used for functional bioluminescenese are algal channelrhodopsins, which are gated by all-trans retinal (ATR) and increase the conductance for monovalent cations across the membrane along their concentration gradient. Rhodopsin ion pumps, in contrast, can also work against an electrochemical gradient and cause unnatural membrane potential variations. The light sensitivity of all channelrhodopsins is governed by the quantum efficiency and extinction coefficient of the covalent ATR group, which is $∼50,000\;M^{-1}\times \;{\mathrm{cm}}^{-1}$.^88^ Differences in their operational light sensitivity or photocurrents, therefore, stem from their single channel conductance, openstate life time, or cell surface expression. The probability of channel gating after photon absorption of ChR is 0.5,^89^ which is conceptually different from the quantum yield [probability that the absorption of a photon causes fluorescence is $\approx 10^{-5}$ (Ref. 90)]. Wildtype ChR2 is a nonspecific cation channel with a preference for H^+^ at physiological conditions. The most common mutant, H134R, shows increased Na^+^ conductivity and retinal binding.^89^ Other mutations shift selectivity to calcium or chloride, or change their action spectrum, whereas these engineered variants are unmatched by naturally occurring channels in terms of their performance.^91^ Considering that bioluminescent enzymes are very weak emitters, a high operational sensitivity is required to achieve the strongest photocurrents and thus neuronal depolarization for a given photon budget. Several channels and pumps (Channelrhodopsin-2,^92^ CheRiff,^93^ Volvox Channelrhodopsin,^92^ step-function rhodopsins ChR2-C128S,^94^ and ChR2-HRDC^95^) or red-emitting luciferases (ChRmine^95^) or to suppress neuronal activity (ACR1,^95^ ACR2,^96^ MAC,^97^ and NpHR^98^) have been combined with neurons expressing blue/cyan emitting luciferases. Because the most powerful luciferases emit in the cyan-teal range (470 to 500 nm), red-shifted ChRs such as ChRmine, still produce weak results, despite their high single-channel conductance.
*LOVTRAP and TULIPs* are plant-derived sensors that can be used to couple light sensing to a change in cellular activity.^99^ The prosthetic light-sensing chromophore is a flavoprotein with flavin mononucleotide (FMN) that couples 450 nm light absorption to a conformational change that alters the Kd of a protein interaction, peptide uncaging, allosteric transitions, and many more.^99^ Different receptors have been engineered that bind strongly or weakly in the dark, thus enabling a light-controlled protein–protein interaction. As consequence of the near-perfect match of the FMN absorption spectrum with Nluc, LOVTRAPs have been used as bioluminescent effectors to drive gene expression in various systems.^100^ Due to the low extinction coefficient ($\varepsilon =14,200\;M^{-1}\;{\mathrm{cm}}^{-1}$), the effective light sensitivity is lower than that of ATR in channelrhodopsin. The operational light sensitivity, however, can be tuned by prolonging its relaxation dynamics after the light is switched off.^101^ An additional benefit of using light-oxygen-voltage (LOV)-domains is that Flavin-binding proteins have a high fluorescence quantum yield and can thus serve as a proxy for protein localization.

### 3.1 Luminopsins

In one of the first attempts to overcome the light delivery problem, light-sensitive ion channels (channelrhodopsins, Box 2) were fused to light-generating luciferases with a spectral overlap of the light emission with the action spectrum of the channel.^92^ These fusion proteins were subsequently coined luminopsins, for a self-illuminating channelrhodopsin [Fig. 3(c)]. The central idea behind this method is to bring the two components into BRET distance, such that the excited state of the luciferase is translated directly into channel gating. Hence, exposing the luminopsin expressing cell to CTZ converts the light-gated ion channel into a designer receptor exclusively activated by designer drugs (DREADD) ion channel, essentially reducing the light delivery to a drug delivery problem. As a starting point, luminopsins consisted in the fusion of the wild-type *G. princeps* luciferase (GLuc) and the classical channelrhodopsin ChR2 (luminopsin 1, LMO1) or channelrhodopsin 1 from *Volvox carteri* (luminopsin 2, LMO2) which produced higher photocurrents at 470 nm.^92^ In the proof-of-principle demonstration, luminopsins elicited subthreshold depolarizations in cultured hippocampal neurons and sensitized them to fire more action potentials^92^ when they were exposed to CTZ.

Ever since, several improvements have been introduced that take advantage of luciferases with higher photon output and/or rhodopsins with different kinetics and ion selectivity, leading up to highly effective tools that enable non-invasive neuronal control.^102^ A fusion of the slow-burn *Gaussia* luciferase (sbGluc^103^^,^^104^) with channelrhodopsin 2, termed luminopsin-3 (LMO3^97^), elicited action potentials in hippocampal neurons after perfusion with CTZ and increased the firing rate in awake and freely behaving mice. A further improvement was achieved by coupling the luciferase to the stabilized step function channelrhodopsin sSFO, [ChR2(C128S/D156A)^105^], which increased the operational light sensitivity of the luminopsin thanks to the long open state lifetime. This resulted in prolonged neuronal activity and superior performance compared with LMO1.^94^ Due to their modular nature, luminopsins can also be used to silence and suppress neuronal activity by fusing the luciferase to an inhibitory instead of an excitatory opsins. Two different, spectrally independent inhibitory luminopsins have been developed consisting either of a red-shifted Nano-Lantern (Rluc8 fused to mVenus^34^) fused to the NpHR light-driven chloride pumps (iLMO2^98^) or the blue-shifted sbGluc moiety to the MAC proton pump from *Leptosphaeria maculans* (iLMO^97^). The functionality of the two inhibitory luminopsins was demonstrated *in vivo*, suppressing spike firing in pyramidal cells of CA3 and CA1^98^ in the hippocampus of anaesthetized rats and in the substantia nigra pars reticulata in living mice with noticeable consequences on contraversive circling behavior.^97^ In contrast, mice expressing the excitatory LMO3 in the same hemisphere circled ipsilaterally.

Due to hardware-independent light delivery, luminopsins hold significant promise for clinical applications. Notably, inhibitory luminopsins have been demonstrated to suppress the amplitude of chemically induced epileptic seizures and reduce their duration in a rodent model.^106^ Likewise, excitatory luminopsins were successfully applied to restore functions in motorneurons after spinal chord injury^93^^,^^107^ and peripheral nerve injury.^108^ It was found that functionally ineffective neurons below the side of injury could be reactivated, resulting in improved spinal circuit function and partial recovery.^107^ In another work, induced pluripotent stem cells transgenic for excitatory luminopsins were used to guide recovery of ischemic brain after stroke.^109^ After stroke, mice that underwent LMO3-iPS-neural precursor cell transplantation exhibited activity-dependent advantages when subjected to CTZ stimulation of these cells. These benefits encompassed the establishment of synaptic connections, enhanced axonal growth and myelination, the development of functional neuronal pathways, increased neuronal plasticity, and notable improvements in functional and behavioral recovery post-stroke. These results demonstrate that luciferase-channelrhodopsin functional coupling can successfully be employed to overcome the challenges related to light delivery before clinical trials can commence in the human brain.^87^ It is worth noting, though, that the application of luminopsins *per se* cannot overcome spatial discontinuities and signaling between cells, as the luciferase and the channelrhodopsin are fused and thus being expressed together on the same cell.

### 3.2 Synaptic Transmission with Photons as Neurotransmitters

**Fig. 4:**
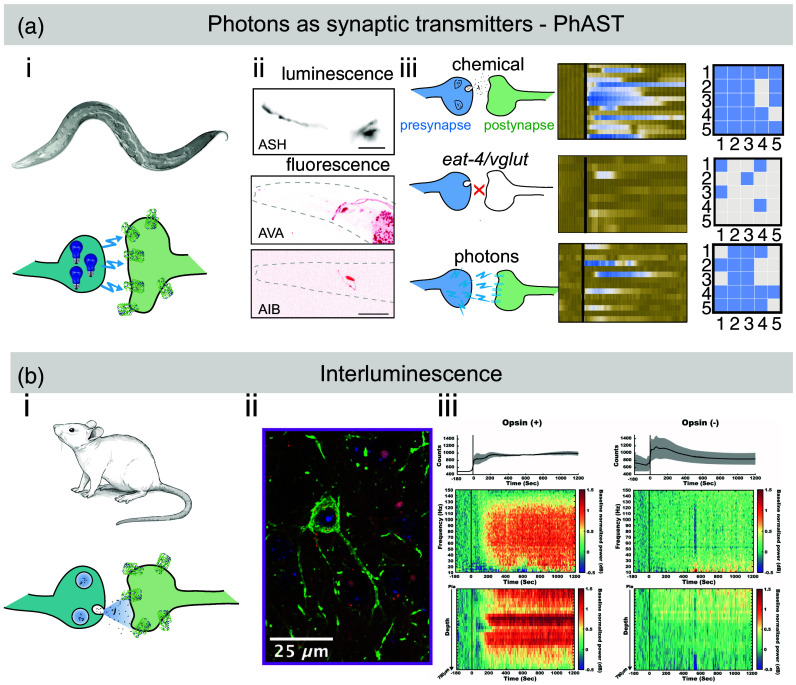
Functional bioluminescence optogenetics through coupling the light output of the luciferase to channelrhodopsin. (a) PhAST was developed in *C. elegans* and (i) consists of soluble but calcium sensitive eNLs that activate their photon emission through their calcium binding domain. (ii) The presynaptic luciferase and postsynaptic channelrhodopsin (ChR2-HRDC) can be encoded with single cell specificity and thus afford circuit-specific investigations. Black shows luminescence from the presynaptic neuron ASH. Red shows representative image of an animal expressing jRGECO1a calcium indicator in the postsynaptic AVA and AIB neurons. (iii) Sketch of the synaptic interaction in wildtype chemical transmission (top), in a synapse of a vesicular glutamate transporter mutant (middle), and a synapse in which chemicals have been replaced by photons (bottom). The kymograph shows the calcium recording of the wildtype (top), vesicular glutamate transporter mutant (middle), and its rescue with photons as synaptic transmitters (bottom). The raster plot shows the behavioral response to five mechanical stimulations of the presynaptic neuron in five animals of the corresponding interventions. Adapted with permission from Ref. 95. (b) *Interluminescence*. (i) Vesicular luciferases that become secreted and activate postsynaptic channelrhodopsin. The system was demonstrated in mice. (ii) Confocal image of a parvalbumin+ neuron expressing the excitatory SFO::YFP (green) along with thalamocortical axon terminals expressing sbGluc::tdTomato (red) and the nucleus in blue. (iii) The top graph shows the average bioluminescence signal with ±1 SEM in presence or absence of channelrhodopsin, while the lower two maps indicate the gamma band activity. Reproduced with permission from Ref. 96.

Inarguably, both repairing damaged neural circuits and gaining control over the wiring of the brain could lead to a variety of new clinical interventions, improved brain function and scientific insight. In an effort to overcome signaling barriers in neuronal networks and to expand the natural repertoire of neuronal signaling, luciferases have been used to cell-autonomously activate channelrhodopsin at a synaptic partner neuron, demonstrating that photons can be used as synaptic transmitters (Fig. 4). Two different systems have been established, consisting of the functional interaction of a postsynaptic channelrhodopsin with photons emitted either from calcium-dependent presynaptic luciferases^95^ or from secreted constitutively active luciferases.^96^ Transsynaptic activation of a photosensitive channelrhodopsin was established in the nematode *C. elegans*, taking advantage of the known wiring connections and previous functional characterization of the nocifensive nose touch circuit.^110^ In this work, controlled and stimulated emission of photons from a presynaptic enhanced mTurquoise Nano-Lantern (TeNL) was used to overcome the behavioral defect of a synthetic glutamate defect that disconnected functional neurotransmission between a single sensory neuron (named ASH) and its cognate postsynaptic interneurons (named AVA and AIB) [Fig. 4(a)].^95^ The calcium sensitivity of the luciferase ensured that light emission occurred primarily when the pre-synaptic calcium concentration was high, e.g., during neuronal activity. This synchronized the photon emission precisely to endogenous events, while preventing the rapid depletion of the substrate that may take place with unregulated, constitutively active luciferases^1^ and/or the inactivation of the enzymatic activity due to rapid catalysis.^40^ To demonstrate the versatility of PhAST, the concept was applied to different circuits in *C. elegans* aiming to suppress the endogenous pain response using a green-tuned inhibitory anion channelrhodopsins (ACR1) coupled to a green-emitting eNL and to rewire a circuit mediating the attractive behavior into an avoidance response.^95^

In another method, termed *Interluminescence*, presynaptically secreted sbGluc functionally interacts either with a step-function opsin ChR2-C128S or the inhibitory anion channel ACR2,^96^ enabling *in vivo* enhancements of brain activity in living mice through the requirement of presynaptic vesicle release [Fig. 4(b)]. To validate the mechanism, *Interluminescence* was also shown in 2D neuronal cultures and depended on intact synaptic connections and vesicle release.^96^ Despite the conceptual similarities of PhAST and *Interluminescence*, both implementations establish a functional connection through different mechanism. In the *Interluminescence* approach, slow burn *Gaussia* luciferase is packaged into synaptic vesicles using a signal peptide from the human pro-opiomelanocortin pro-peptide (hPOMC1-26::sbGluc), which become secreted into the synaptic cleft upon successful synaptic activation. Once released, the sbGluc diffuses across the cleft and activated postynaptic channelrhodopsins to elicit a downstream neuronal response. *Interluminescence* appears to indicate optical signaling occurring at functional synapses due to the requirement for the close proximity of luciferase and postsynaptic opsins within a shared synaptic cleft. The occurrence of *Interluminescence* beyond established synapses seems improbable, although this potentiality could be subject to future investigation.^96^ In addition, the absence of a postsynaptic response to luciferin added 20 s after release suggests that luciferases diffusing away from the synaptic space likely lack a sufficient photon density to activate opsins along the neuron. Because *Interluminescence* requires vesicle release at primarily synaptic sites, *de novo* generation of asynaptic connections needs to be further demonstrated. In PhAST, the luciferases are distributed throughout the neuron and may not necessarily be confined to the presynapse (unless specifically concentrated there using synaptogyrin tags^95^), and thus, the transient photon emission might activate neighboring neurons expressing channelrhodopsin in a fashion similar to neurotransmission.

Both techniques share conceptual similarities such that PhAST and *Interluminescence* required postsynaptic channelrhodopsins with an extended open state lifetime, which might be limiting the temporal dynamics to regulate fast-switching synaptic activity. Both are also highly modular and the light emitted by the luciferase can either activate or inhibit proximal neurons based on the expressed opsin, presenting an advantage compared with methods needing distinct systems for both activation and inhibition. The wavelength of the emitted photon can be coupled to different opsins and thus facilitates the design of more complex networks with multiple luciferase-opsin connections.^95^ Furthermore, PhAST and *Interluminescence* employ rhodopsins as current carriers, directly influencing alterations in the membrane potential of the postsynaptic partner, in contrast to other synaptic engineering techniques that leverage G-protein-coupled receptor (GPCR) signaling pathways or calcium ion flux.^3^ Finally, PhAST has the advantage that the light output increases with presynaptic activity and, in principle, these photons can be imaged on a camera detector and can thus also be purposed as an indicator for synaptic activity.^3^^,^^12^

Taken together, PhAST and *Interluminescence* represent two powerful approaches to achieve synapse-specific and activity-dependent circuit control *in vivo* by means of synaptic engineering, and thus complement a toolbox of genetically encoded designer molecules capable of building and rewiring functional neuronal networks.^111^^,^^112^

## 4 Workshop: Optimizing Bioluminescent Signals for *in Vivo* Imaging

### 4.1 Optimizing Expression Levels

Like all genetically encoded sensors, the expression of luciferases can be optimized through the insertion of a multicopy transgene in a transcriptionally active locus. In mice, the Rosa26 locus proved well for stable expression; alternatively viral delivery of transient expression systems can be applied. The latter is faster and more efficient; however, it comes at the expense of varying expression levels. In *C. elegans*, the possibility exists to propagate multicopy transgenes as a metastable array or even integrated in safe harbor loci.^113^ Further, the signal of luciferase emission can be optimized using mutants with increased thermal stability,^114^ expression levels can be optimized by codon adaptation of the cDNA to the host organism, while the signal/background ratio can be increased with the use of targeting sequences that enrich the luciferases at certain subcellular structures, e.g., the nucleus or synapses. This was successfully achieved by fusing Syn-ATP to Synaptophysin^4^ or TeNL to SNG-1 synaptogyrin^95^ to enhance the localization of the luciferase at presynaptic varicosities in mouse neurons or *C. elegans* ASH neurons, respectively; but also the nucleus in mouse embryonic stem cells.^12^ Codon optimization of *Gaussia* luciferase cDNA sequence has been shown to increase expression levels in the heterologous host compared with its wildtype form,^115^ which became the standard in the field.^24^^,^^95^ Likewise, the insertion of artificial introns is commonly known to increase transgene expression in *C. elegans*^95^^,^^116^ and specific 3’UTRs might regulate expression through mRNA stabilization in a cell-type specific manner.^117^

### 4.2 Cofactor Delivery for Neuronal Imaging

The most common luciferases for use as *in vivo* reporters are Fluc, Rluc, Gluc, and Nluc derivatives. As different luciferases require different cofactors, optimization strategies should strongly depend on the specific enzyme in use. d-luciferin, the firefly substrate, is more water-soluble than the CTZ analogs for Gluc and Rluc, which sets an upper concentration limit that can be applied to cells and tissues.^38^^,^^118^ Further, CTZ has the tendency to oxidize spontaneously, generating spurious *in vivo* signal.^119^ Imaging of neuronal activity in the brain of higher vertebrates poses additional challenges which lie in the delivery of the critical photogenic co-factors and their passage over the blood-brain barrier.^120^ Likewise, the exoskeleton and the cuticle in invertebrates is often encountered as a diffusion barrier that needs to be overcome.^121^^,^^122^ Thus, the bioluminescent signal may not report the cellular activity but the distribution of the cofactor, requiring luciferin delivery optimization for different tissues. In *C. elegans*, we consistently observed bioluminescence in amphid cells (ASH and AWA) or the vulva regions that are exposed to the environment under conditions in which we did not observe signal in other tissues. Among the several chemical modification of existing cofactors that have been introduced over the recent years to both improve their bioavailability and retaining their highly efficient photon emission,^14^^,^^38^^,^^39^^,^^45^^,^^46^^,^^118^^,^^123^ we found that FFz^45^ works exceptionally well in *C. elegans* when animals carry mutations in certain cuticle collagens or crosslinkers that facilitate diffusion across their skin. The *bus-17* or *bus-5* mutations, for example, have been used extensively to facilitate drug delivery to *C. elegans* in other contexts.^12^^,^^124^^,^^125^ Alternatively, micelle or liposome-forming surfactant or coblock polymers can stabilize the hydrophobic cofactors and facilitate passage of drugs across the cuticle.^95^^,^^126^ In general, due to the thick cuticle of adult *C. elegans*, incubation at 10 to 100 times the concentration that would be used on isolated cells should be considered. Also the application of the compound at younger larval stages facilitates delivery. Other cofactors that have been shown to increase bioavailability and transport across the blood–brain barrier are CFZ^39^ or ETZ, which stands for an esterase-dependent activation and enhanced *in vivo* performance.^38^ ETZ is a carboxylated form of DTZ with better permeability across the blood–brain barrier that generated red-shifted photons^44^ and afforded transcranial imaging of calcium dynamics in various regions of the brain in anaesthetized or awake mice.

### 4.3 Potential Side Effects and Toxicity During Cofactor Administration

As with any exposure to a chemical, one should consider potential side effects of the luciferin, especially for long term *in vivo* applications. At least two studies reported increased toxicity at high concentrations above 10  μM for furimazine^127^^,^^128^ and 20  μM for CTZ.^129^ This differs from the reports that credit antioxidant characteristics to imidazolopyrazinones,^130^ to which CTZ and furimazine belong. Indeed, several reports found no toxicity of furimazine under similar conditions for concentrations up to 50  μM,^131^^,^^132^ suggesting that the observed toxicity is due to a cell-type specific response. Interestingly, it was found that CTZ is a substrate for the multidrug resistance transporter P-glycoprotein (PGP), which efficiently pumps CTZ out of cells or off the plasma membrane, resulting in decreased intracellular availability of these substrates.^133^ *C. elegans* contains several PGP homologs that protect the animals against natural toxins,^134^ but whether or not the expression of PGPs in *C. elegans* limits substrate availability and bioluminescence remains to be shown. In case where toxicity is a concern, modified furimazine analogs have shown reduced toxicity and increased bioavailability.^39^^,^^45^^,^^128^^,^^135^ Likewise, exposure of living *C. elegans* animals to high concentrations of furimazine does not elicit an immediate stress response, as judged by the absence of nuclear localization of FoxO transcription factors.^12^ The observed differences in toxicity may also stem from the various solvents used to solubilize CTZ and its analogs. Toxic effects of ethanol as a solvent could be prevented using 2-hydroxypropyl-β-cyclodextrin and permitted non-invasive experiments in zebrafish.^11^

While d-luciferin is generally accepted as a non-toxic cofactor, high concentrations and continued exposure may lead to side effects due to ATP depletion within the expressing cell. Components of the bacterial *lux* system may be toxic to eukaryotic cells due to the fatty aldehyde metabolism.^65^ Recent data, however, did not confirm these results.^64^ Likewise, no toxicity was observed in cells expressing components of the fungal bioluminescence pathway that were exposed to hydroxy-hispidin,^136^ which might even be beneficial as an anti-oxidant.^137^

### 4.4 Every Photon Counts—Optimizing Light Collection for 3D Bioluminescence Microscopy

Due to the slow turnover and reaction cycle, luciferases are intrinsically photon starved. Despite the background-free signal generation, they require long exposure time to reach acceptable image quality on standard widefield microscopes, from tens of second to the minute scale.^4^^,^^12^^,^^13^ To improve photon collection and decrease exposure time, several strategies have been pursued. Among the components that are most critical to reduce the exposure time are the collection lens and the camera. It is important that the lens collects as many photons as possible emitted toward the microscope. Thus, a higher numerical aperture (NA) almost always leads to more efficient photon collection. However, the trade-off lies in the magnification of the objective, which distributes the photons over more pixels and thus decreases the SNR. Pixel binning with a scientific complementary metal-oxide-semiconductor (sCMOS) camera does not necessarily improve conditions for very faint signals close to SNR limit, as the summation during the binning operation is performed after readout (and thus adds 4× readout noise for 2×2 binning), whereas in charge-coupled device (CCD) cameras, it is hardware-based (1× read out noise irrespective of binning). To maximize brightness and contrast, we compared a Nikon 40×/1.15 WI and an Olympus 40×/1.25 silicon immersion (SIL) objective and collected significantly more photons with the former under the same excitation conditions. This is not surprising due to the higher NA and better refractive index match of the silicon immersion oil with biological media. As photons are sparse and limited, the photons should ideally not spread out over many pixels and unnecessary magnification should be avoided (e.g., a slight increase in NA is often counterproductive if the magnification also increases). Thus Nyquist sampling is important, or if resolution can be sacrificed, slight undersampling will improve the SNR dramatically—this is commonly achieved by demagnifying the image with a 0.5× tube lens.^12^^,^^13^^,^^138^ Consequently, as no filters and mirrors are required in self-illuminating samples, the optical axis can be built extremely short and quasi-lossless.

**Fig. 5:**
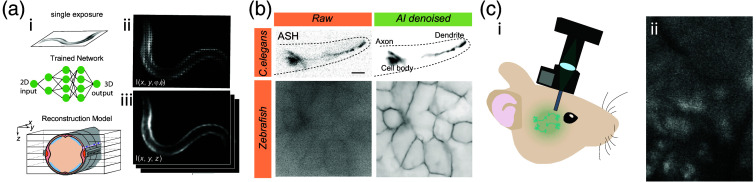
Advances in microscopy and image processing for bioluminescence imaging. (a) (i) Schematic of LFM approach for subsecond volumetric imaging of calcium activity in freely moving animals. A single 2D camera exposure with four-dimensional information can be reconstructed to obtain a 3D representation of the bioluminescent scene using properly trained neural networks. (ii) Raw light field image of an animal expressing calcium sensitive TeNL in body wall muscles. (iii) Reconstructed x,y planes after deep learning deconvolution. Adapted from Ref. 12. (b) Content aware restoration pipelines based on machine learning can be used to obtain high SNR images from severely degraded images with poor SNR. Adapted from Ref. 12. (c) The excitation-free imaging in bioluminescence microscopy affords smaller head-mounted miniscopes for neuronal calcium imaging in freely behaving rodents. The miniscope does not require excitation LED, meaning less energy consumption. Excitation and emission filters can be omitted and the overall reduction weight is less invasive and permits long term imaging. (i) Sketch of the system and (ii) representative image of a bioluminescent mouse brain after CTZ administration. Adapted from Ref. 140.

The second most critical component is the camera. Modern sCMOS cameras have >95% quantum efficiency and a dark current well below 1 electron/pixel/s and with appropriate cooling 0.2 electron/pixel/s with ≈1 electron rms read noise. Because the lower dark noise of electron multiplying (EM) CCD cameras is only an advantage for long exposure times in the tens of second or minute scale, we found that back-thinned sCMOS cameras have a better performance on short exposure times. With the new generation of quantitative CMOS (qCMOS) cameras and their ultra-low readout noise (0.24 electrons) and dark current (0.006 electrons/s/pixel), single photon events can be resolved and quantified on a pixel-by-pixel basis without electron multiplying excess noise. At the same time, the qCMOS camera has a pixel size that is 3 to 4× smaller than that of an EMCCD, which is critical to achieve good spatial sampling in advanced techniques such as light-field microscopy (LFM). LFM is a plenoptic imaging technique that leverages a microlens array to capture both the intensity and direction of light rays from a sample,^139^ allowing the reconstruction of three-dimensional images from a single photographic exposure [Fig. 5(a)]. As only a single exposure is required, it is a promising way to obtain volumetric information from a bioluminescent sample with just a few captured photons, albeit with the trade-off in spatial resolution. Recently, we demonstrated that LFM can be used to image calcium activity in muscle cells of freely moving *C. elegans* with down to 200 ms exposure times [Fig. 5(a)]. Importantly, light field imaging is easy to implement, but the deconvolution is a major bottleneck that limited the widespread adoption of the technique by the scientific community. Several machine-learning-based neural networks are available^141^^,^^142^ that perform reconstructions of the light-field images to generate image stacks, and (after proper training) also provide image resolution beyond the nominal resolution of the lenslet array.^12^

### 4.5 Smart Image Acquisition and Content Aware Reconstruction

Obtaining a visually appealing, high-contrast image may involve trade-offs with physiological accuracy. This can be attributed to issues such as overexpression artifacts, fixation or immobilization of the living animals, the need for intense illumination, and long exposure time, all of which are necessary to capture a sufficient amount of photons for statistical analysis and pleasing visual perception. Even with the improvements outline above, bioluminescence microscopy almost always requires exposure times that are significantly longer than with fluorescence microscopy,^35^ which may be incompatible with swift biological dynamics inherent to living cells, especially when imaging calcium in the brain. Thus, the challenge is to capture just enough photons that are necessary to deduce the information presented in the scene. Further, bioluminescence microscopy is challenging as the whole sample is luminescent and thus lacks optical sectioning in the excitation, thick samples inadvertently generate more out-of-focus blur than, e.g., widefield fluorescence excitation as shown in Fig. 5(b) (because the excitation intensity decreases with the square of distance from the focal plane).

Content aware deep learning models have been used extensively in fluorescence microscopy to minimize phototoxicity [Fig. 5(b)], e.g., by reducing the exposure time or laser power in a microscopy experiment.^143^ Models are trained by processing large amounts of input-ground truth image pairs, while iteratively minimizing a loss function through the application of stochastic gradient descent. During this process, the model refines its internal parameters by adjusting them in the direction that reduces the difference between the ground truth and the model inference. This adjustment is guided using random subsets of the training data allowing the model to efficiently navigate the high-dimensional parameter space. As the training progresses, the model’s performance steadily improves until the loss converges to a satisfactory level, indicating that the model has learned to generalize well to new, unseen data. Properly trained models can be used to remove noise and augment the resolution of a severely photonstarved and undersampled bioluminescence microscopy image to produce a clean image with a high SNR [Fig. 5(b)]. These methods were demonstrated to yield satisfactory improvement of bioluminescence microscopy images in *C. elegans*, mouse embryonic stem cells, and zebrafish,^12^ and afforded image segmentation, cell tracking, and other post-processing pipelines to extract quantitative information that was impossible to obtain without content aware restoration.^12^ Crucially, this approach proved critical to capture 3D calcium dynamics in freely moving worms by LFM, because it afforded (1) a significant reduction of exposure time and (2) an improvement in image quality which simplified the light field reconstruction algorithms.^12^

### 4.6 Limitations of Bioluminescence for Microscopy

Despite the ongoing effort to increase the versatility of luciferases and their cofactors as bioluminescent sensors and reporter systems, the technique has some important limitations. First, the catalytic turnover of the most common enzyme, firefly luciferase, is slow with ≈1.6 cycles per seconds.^144^ While current Nluc derivatives have higher catalytic turnover rate, it is still a factor of >10 dimmer than FPs. This limitation poses obstacles when applying it as a fusion tag for proteins with low abundance or as a reporter for rapidly changing dynamics (such as calcium dynamics or voltage imaging), necessitating further improvements in brightness.

As discussed extensively in this primer, bioluminescence imaging requires a chemical substrate rather than external light for excitation. The supply and consumption of this substrate can limit imaging. While most marine luciferases do not require ATP for their reaction, ensuring a constant supply of the substrate remains necessary. Clever perfusion and or conditionally active luciferases may improve long-term imaging, as well as luciferases with limited self-inactivation. Likewise, the oxidation of the luciferin most commonly requires oxygen, which may become a rate-limiting factor in deep tissues that are under metabolic demand, such as the brain or tumors.^145^ Thus, careful evaluation of the data and/or the use of oxygen-independent luciferases (e.g., from bacteria) may be applied.

Bioluminescence imaging lacks optical sectioning capabilities. Unlike FPs that emit signals only when exposed to excitation light, luciferases emit signals throughout the sample. As a result, signals from luciferases out of the focal plane contribute to a haze in thick samples (Fig. 5). This limits the signal background ratio. To mitigate this problem, specific transgenic methods can generate sparse labels, or deep learning techniques can be applied to facilitate image restoration.

Multicolor imaging using different luciferases emitting different colors can be performed, although their emission is usually quite broad and their spectrum might overlap significantly. Separating different colors can solely be done using emission filters, and significant channel bleedthrough is expected. This necessitates the use of a linear unmixing algorithms or advanced phasor analyses to achieve better separation.^132^

Even though red-shifted luciferases are popular among neuroscientists for their improved tissue penetration, they might be suboptimal for high-resolution imaging. This is because the spatial resolution limit d depends on the wavelength λ of the captured light, which is according to the Abbe limit $d=\frac{\lambda }{2\mathrm{NA}}$, with NA as the numerical aperture of the objective. Thus, a luciferase emitting at 450 nm affords a 100 nm higher resolution than one emitting at 700 nm for the same NA = 1.3 objective lens. Likewise, superresolution in many fluorescence modalities is often achieved by point-spread engineering of the excitation light - which is absent in bioluminescence microscopy. Thus, conceptually different modalities need to be conceived in order to break the diffraction limit in bioluminescence microscopy.^146^

Finally, there is an almost impenetrable complexity and variety of different bioluminescence systems available with various luciferases and their mutants, in combination with chemically engineered cofactors. Many of these systems have been extensive characterized *in vitro* with detailed information about the quantum yield and catalytic turnover available. Their translation into *in vivo*, however, requires knowledge about the bio-availability (distribution, stability, and transport) and toxicity of the cofactor, and how the emitted photon interact with the surrounding tissue. A direct, unbiased comparison of the most promising luciferases and their cofactors in the same cellular environment or animal model would provide valuable insights into their mode of action.

## 5 Future Directions

### 5.1 Combination of Luciferases, Synthetic Photoswitches, and Metabotropic Photoreceptors

The use of photons as synaptic transmitters is also not limited to ionotropic channelrhodopsins. The use of metabotropic rhodopsins would allow for a multiplicative photoresponse as compared with the linear photoresponse of channelrhodopsins or sublinear response of ion pumps. In this scenario, photosensitive GPCRs can be expressed in heterologous systems and hijack endogenous G signaling proteins, which leads to amplification of the signal as each active G-protein produces multiple active second messengers.^147^ For that to work, the precise knowledge of the endogenous G-protein repertoire, however, needs to be taken into account, to avoid cross-reactivity. In an alternative approach, endogenous metabotropic GPCRs can be rendered light sensitive by genetic code expansion and chemical conjugation of a photo-responsive group.^148^^,^^149^ These approaches are again not limited to classical ionotropic and metabotropic neuromodulation but can, in principle, apply to any light-activated receptor to trigger intracellular signaling cascades^150^^–^^152^ that modify the cytoskeleton, apoptosis, or neurogenesis. However, many of these tools require high illumination intensities to activate the photosensitive protein (Box 2) or photons in the violet spectrum to trigger the conformational changes of a photoswitch. Either new high-power luciferases emitting in the UV or alternative photoswitches are needed.^153^

### 5.2 Combination of Calcium-Dependent Emission in the Brain and Diffuse Optics

Bioluminescence tomography (BLT) is a powerful, pre-clinical method to localize a light source within a 3D tissue by applying principles of diffuse optical spectroscopy to bioluminescent emitters.^154^ It aims to perform quantitative 3D reconstructions of internal light sources from different angular perspectives measured on the external surface of the animal, e.g., mouse brain. In contrast to planar bioluminescence imaging, it is based on a model for light propagation in optically diffuse, biological tissues, taking scattering and absorption into account and thus provides a quantitative readout for intensity, size, morphology, and dynamics.^154^ Even though it was primarily applied to monitor tumor growth and metastatic spreading, BLT holds promise in neuroscience as it provides information about source intensity and localization in 3D. Thus, the emergence of red-shifted powerful, calcium-dependent luciferases, paired with BLT may afford spatially resolved, functional imaging in the brain of model animals. However, due to the high-scattering properties of the brain and photon absorption by tissue chromophores such as melanin, hemoglobin, and other proteins, quantitative description of source intensity and localization precision is usually limited to ≈3  mm.^154^^,^^155^ The application of advanced models and deep neural networks may be a solution to increase the versatility for multisource reconstruction and have been shown to reduce the localization error down to ≈1  mm.^155^^,^^156^

### 5.3 Bioengineering: Autonomous Luciferase/Luciferin Systems and Faster Luciferases

Since the first report of the small and bright Nluc^28^ luciferase, more than 30 different Nluc derivatives have been established as fusion proteins with solute and metabolic sensors (Fig. 2). The Nluc moiety, however, has been left relatively untouched with only a few published studies reporting different modifications and their effects.^26^^,^^44^ As outlined in the original report, factors limiting the photon output are the enzyme substrate turnover and the quantum yield.^28^ While the quantum yield has been partially increased by fusing the luc moiety to different FPs with diverse outcomes,^22^^,^^25^^,^^35^ substrate turnover remained relatively unaltered. While existing luciferases may be tuned in their conformational flexibility to increase substrate binding and release kinetics,^157^ a different and challenging approach may constitute the complete and *de novo* synthesis of an unnatural luciferase^158^ with superior properties.

Another factor that is poised for improvement is the possibility of synthesizing the cofactor directly inside the cell of interest. While the metabolic pathways leading to CTZ and d-luciferin are still being worked out,^159^ the synthetic pathway for bacterial^160^ and fungal^136^ luciferins has been reconstituted in heterologous cells. As previously mentioned, the bacterial lux system was applied to *C. elegans*^64^ and tissue culture cells,^161^ among others, where it sustained long term bioluminescence recordings. On the other hand, the luminiscent system from the poisonous mushroom *Neonothopanus nambi* poses an alternative, promising strategy. It requires two enzymes to produce hydroxy-hispidine (fungal luciferin) from caffeic acid and two more enzymes (the Luz luciferase and a phosphopantetheinyl transferase to posttranslationally modify and activate it) are required to oxidize it and emit light.^136^ This system has been successfully engineered into transgenic plants and yeast cells, producing luminescence visible to the naked eye.^136^^,^^162^^,^^163^ One additional enzyme can be employed to reconstitute hydroxy-hispidine from its oxidized form and thus increase bioluminescent efficiency,^136^ while three additional enzymes can be encoded to produce caffeic acid from either tyrosine^136^ or phenylalanine^162^ and produce autonomous luminescence in organisms lacking caffeic acid biosynthesis. Even though the *N. nambi* luciferase has been proved functional in transgenic plants, yeast, frogs, and mice, the reconstitution of the biosynthetic pathway in cultured neurons or whole animals has to be worked out. The successful implementation of this pathway would be a great step toward overcoming the challenge of administered cofactors *in vivo* and would enable the possibility of long-term imaging and functional interrogation in a clinical model.

### 5.4 Advances in Handheld Bioluminescence Imaging for *in Vivo* Applications

Since the introduction of integrated portable microscopes, known as miniscopes,^164^ significant strides have been made in neuroscience, particularly in understanding cognitive and social behavior, memory, and olfactory^165^ processing across various biological models. However, these devices still face limitations associated with the need to project light onto the brain, inheriting challenges from fluorescence imaging. These issues include interference from autofluorescence in native tissues and non-homogeneous illumination due to light scattering during excitation.

To address these limitations, the concept of a bioluminescence miniscope (BLmini) has been proposed,^140^ relying entirely on the natural emission of light from the specimen, eliminating the requirement for external excitation light optics [Fig. 5(c)]. Consequently, the BLmini offers enhanced sensitivity, reduced weight, power consumption, and simplified assembly compared with traditional fluorescent miniscopes. Moreover, the elimination of optical components required in fluorescence microscopy leads to a reduction in the overall light loss along the emission light path. However, its potential for calcium imaging has not been explored, and in the context of extended imaging experiments, fluctuations in SNR may occur due to the rapid decay of bioluminescent signals or the dependency of oxygen for the photogenic reaction.

Alternative devices have been introduced based on consumer product, such as a handheld, smartphone-based camera detection to monitor bioluminescence *in vitro*,^166^ from organelles to whole animals.^167^ The widespread use of these devices might be particularly important to reach the transition from the bench to the bedside and facilitate the use of bioluminescence as diagnostic tools.

The successful implementation of these techniques could pave the way for the development of computational pipelines that maintain consistently high SNR imaging and investigate their capacity and robustness in obtaining precise quantitative data on neuronal activity. Moreover, head-mounted miniscopes can be integrated with the light field imaging methods^12^^,^^168^ to obtain 3D information with a single exposure, amplifying their potential and broadening the scope of biological insights they can provide. Such techniques would enable digital refocusing, stereo visualization, as well as surface and depth mapping of microscopic scenes in freely moving animals. However, as the extended depth of field of the LFM approach results in a sacrifice of spatial resolution, the combination with alternative approaches, including holographic super-resolution techniques such as Fresnel incoherent correlation holography (FINCH)^146^ or adaptations of LFM such as Fourier LFM,^169^ holds a great promise in enabling single-shot imaging for higher resolution, potentially unveiling crucial subcellular details in bioluminescent studies.

## 6 Conclusion

Here, we have provided a primer for the use of luciferases for *in vivo* imaging of physiological processes and to obtain cellular control through functional bioluminescence optogenetics. We highlighted recent developments in cofactor chemistry and bioengineering to convert naturally occurring luciferases into suitable biosensors for *in vivo* applications. We conclude that current efforts are focused primarily on improving Nluc varieties for their powerful photon output and their small size. However, the majority of the application of these sensors remain exploratory and have been used solely as proof of principle probes to image calcium activity in neurons under external interventions, but lack the widespread adoption by the scientific community, primarily due to the old stigma of being “not bright enough.” Nevertheless, the applications of bioluminescence in the brain are expanding in the hands of bioengineers and neuroscientists. Over the recent years, this opened the new field of functional bioluminescence optogenetics, which aims to build a genetically encoded, all-optical interface to overcome the light delivery challenges in classical optogenetics, and to control neuronal activity by coupling the light output of the luciferases to genetically encoded photosensitizers. We forecast that the combination of new developments in microscopy and photon detection, together with continued efforts in bio- and chemical engineering to further improve light output and wavelength selectivity promise a bright future for bioluminescence in neuroscience.

## Data Availability

No new code or data were produced in this manuscript.
